# Acupuncture-Associated *Mycobacterium massiliense* and *Scedosporium* Infections Superimposed by Tetanus

**DOI:** 10.1155/2022/8918020

**Published:** 2022-07-07

**Authors:** Thaninee Prasoppokakorn

**Affiliations:** Division of Gastroenterology, Department of Medicine, Faculty of Medicine, Chulalongkorn University and King Chulalongkorn Memorial Hospital, Thai Red Cross Society, Bangkok, Thailand

## Abstract

**Background:**

Tetanus has rarely been reported in Thailand since 1982 due to 100% tetanus vaccination coverage during the neonatal period of life. However, the reemergence of tetanus has been observed in our country during the past decade, mainly due to the increasing number of migrants traveling from neighboring countries in search of work. Acupuncture has become an essential part of alternative and complementary medicine. To our knowledge, acupuncture-associated *Mycobacterium abscessus* and *Scedosporium* infections superimposed by tetanus have never been reported. *Case Presentation*. A 55-year-old Thai female with schizophrenia was hospitalized due to a 4-day course of trismus, dysphagia, and back muscle spasms. Upon admission, a clinical diagnosis of tetanus was made, which included muscle rigidity and reflex muscle spasms, despite a recent history of diphtheria-tetanus (dT) vaccination for tetanus prophylaxis after 2 episodes of falling complicated by two lacerations on the left shoulder and head. Endotracheal intubation for airway protection was given, in addition to tetanus immunoglobulin, metronidazole, and diazepam which were prescribed to the patient. Incision and drainage of the wound on the left shoulder yielded 40 mL of pus, which subsequently grew *Clostridium* species, *Mycobacterium massiliense, and Scedosporium* on anaerobic bacterial, mycobacterial, and fungal cultures, respectively. An incision of an acupuncture wound on the abdominal wall yielded 1 mL of pus, which exhibited positive acid-fast bacilli (AFB) on AFB stain. Mycobacterial culture finally grew *M. massiliense*. The organism was susceptible to amikacin and clarithromycin. Amikacin, clarithromycin, ciprofloxacin, and voriconazole were then added. The patient gradually improved and was discharged after one month of hospitalization. The patient was reported to be doing well, with no neurological sequelae, when last seen one month after discharge.

**Conclusions:**

To our knowledge, this is the first case of acupuncture-associated *M. massiliense* and *Scedosporium* infections superimposed by tetanus. In Thailand, the occurrence of acupuncture by nonqualified individuals and the reemergence of tetanus remain prevalent. Hence, it is not uncommon to see tetanus in association with acupuncture-related nontuberculous mycobacterial/fungal infection.

## 1. Introduction

Tetanus is an acute neuromuscular disease caused by the anaerobic bacterium *Clostridium tetani* [1, 2]. Since 1982, it has rarely been reported in Thailand due to 100% tetanus vaccination coverage during the neonatal period of life. However, the reemergence of tetanus has been observed in Thailand during the past decade, mainly due to the increasing number of migrants traveling from neighboring countries in search of work. Furthermore, reported national survey of tetanus antibody in the Thai population demonstrated lower titer of antibody in the elderly group especially in those who had sustained any tetanus-prone injury [3].

Acupuncture has become an essential part of alternative and complementary medicine [4]. Although exact epidemiological studies of acupuncture in Thailand are lacking, there remains reported increase of traditional medication and acupuncture in the public health service system [5]. Additionally, in Thailand, it tends to be performed by unauthorized organizations including beauty salons, massage parlors, barbershops, and clinics, as well as nonqualified individuals. Consequently, acupuncture-associated infections are not uncommon in our country. To our knowledge, acupuncture-associated *Mycobacterium abscessus* and *Scedosporium* infections superimposed by tetanus have never been reported.

## 2. Case Presentation

**Figure 1:**
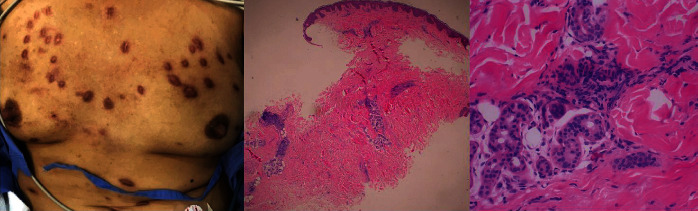
Multiple, discrete, firm, smooth, skin-colored, and hyperpigmented papules as well as plaque on the upper chest, abdomen, and both knees (left). Haematoxylin and eosin stain histopathology of a skin biopsy (middle) using low power field revealed perivascular and peri-adnexal lymphohistiocytic infiltration in the deep dermis, while (right) high power field revealed periductal mixed inflammatory cell infiltration, representing a dermal inflammatory reaction.

**Figure 2:**
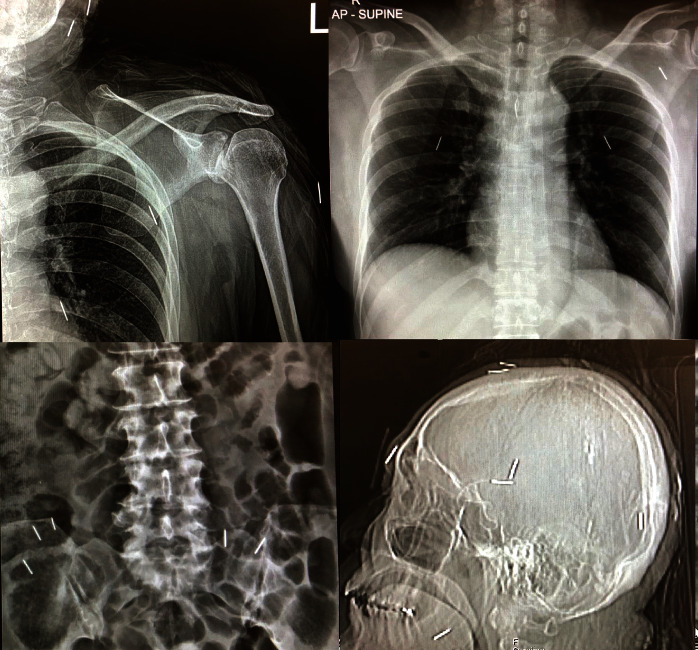
Plain X-ray exhibiting many 1 cm pin-like metallic opacities at the posterior chest wall, paraspinal area of the lumbosacral spines, and skull.

A 55-year-old Thai female was hospitalized at King Chulalongkorn Memorial Hospital, Bangkok, Thailand, on December 2018, due to a 4-day course of trismus, dysphagia, and muscle spasms in the back following 2 episodes of falling, which were complicated by 2 lacerations on the left shoulder and head sustained 2 weeks and 1 day prior to the present illness. The patient received primary wound closure, oral dicloxacillin, and diphtheria-tetanus (dT) vaccine after the second fall. Her past medical history was unremarkable except for a recent diagnosis of schizophrenia and a history of body acupuncture with 41 needles being pierced into her whole body by an nonregistered acupuncturist 2 years ago. The motivation for acupuncture was her faith in a medium's ability to communicate with spirits. However, the acupuncture technique in this case was performed illegally by a nonregistered individual with wide variation in different illegal places. We were not able to obtain detailed information concerning the procedure. Her vaccination status could not be recalled. The result of anti-HIV testing was negative. Upon admission, physical examination revealed body temperature of 38.5°C, blood pressure of 130/80 mm·Hg, pulse rate of 110/min, respiratory rate of 22/min, two recent lacerations on the posterior region of the head and signs of inflammation with area of fluctuation on left shoulder, as well as numerous hyperpigmented, hypertrophic scars on the head, abdominal wall, and back that appeared to be old. A neurological examination revealed generalized muscle rigidity, including spastic dysarthria, trismus, and opisthotonos, as well as reflex spasms comprising blepharospasms and risus sardonicus. An initial laboratory investigation was unremarkable except leukocytosis. A clinical diagnosis of tetanus was made, after which the patient was promptly given endothecheal intubation, tetanus immunoglobulin (TIG), metronidazole, and diazepam. In addition, incision and drainage of the wound on the left shoulder were performed, which yielded 40 mL of pus exhibiting Gram-positive bacilli on Gram stain and positive acid-fast bacilli (AFB) on AFB stain. Anaerobic bacterial, mycobacterial, and fungal cultures consequently grew *Clostridium* species, *Mycobacterium massiliense, and Scedosporium,* respectively. Minimum inhibitory concentration (MIC) of *M*. *massiliense* using the Sensititre Vizion™ System (TREK Diagnostic Systems, MA, USA) is shown in Table 1. An incision in the acupuncture wound on the abdominal wall yielded 1 mL of pus, which exhibited positive acid-fast bacilli (AFB) on AFB stain. In addition, a dermatologist was consulted to evaluate the skin lesions, described as multiple, discrete, firm, smooth, skin-colored, and hyperpigmented papules, as well as plaque on the upper chest and abdomen representing keloid infection. A skin biopsy was performed. Histopathology exhibited perivascular and peri-adnexal lymphohistiocytic infiltration in the deep dermis, representing a dermal inflammatory reaction (Figure 1). Mycobacterial culture from abdominal pus finally grew *M. massiliense*, which was confirmed by PCR and line probe assay to identify the species of *Mycobacterium*, even though mycobacterial blood cultures cultivated no organisms. Plain X-ray showed many 1 cm pin-like metallic opacities along the posterior region of the chest wall, paraspinal area of the lumbar spine, and skull (Figure 2). Intravenous amikacin, oral clarithromycin, ciprofloxacin, and voriconazole were then added. The dose of diazepam was increased to the maximum of 120 mg/day to control muscle rigidity and reflex spasms. The patient gradually improved and was discharged one month after hospitalization when dT was vaccinated. The complete 6-month course for *M. massiliense* was prescribed. She was doing well without neurological sequelae when last seen one month after discharge.

## 3. Discussion

To the best of our knowledge, this is the first case of acupuncture-associated *M. massiliense* and *Scedosporium* infections superimposed by tetanus.

Tetanus is a life-threatening but preventable infectious disease caused by the tetanospasmin of *C. tetani*. The incubation period ranges from 2 to 21 days. A diagnosis of tetanus is made clinically with the inclusion of generalized muscle rigidity and reflex muscle spasm [1]. In our patient, we think tetanus was acquired after the first injury at her shoulder 2 weeks prior to her present illness since she did not receive adequate wound care and dT vaccination. However, the anaerobic cultures of the pus obtained from the wound at her shoulder did not grow *C. tetani,* which is consistent with many previous reports showing only 20–30% of organism isolated [1]. Vaccination with dT after the second injury 1 day prior to her present illness will probably modify the course of tetanus to the easily controllable form of tetanus [2].

Traditional needle acupuncture, a component of alternative medicine, has been gaining popularity worldwide, especially in China and Southeast Asia as in our country [4]. To date, acupuncture-associated infections have increasingly been reported. Most infections are caused by pyogenic bacteria, nontuberculous mycobacteria especially rapidly growing mycobacteria (RGM), and fungi [4]. RGM causing acupuncture-associated infections include *M. abscessus, M. massiliense* (formerly *M. abscessus* subspecies *massiliense*)*, M. bolletti* (formerly *M. abscessus* subspecies *bolletti*), and *M. fortuitum.* Acupuncture-associated infections caused by *M. massiliense* have rarely been reported. However, the recent taxonomic classification suggested *M. abscessus* subspecies *abscessus, M. abscessus* subspecies *massiliense,* and *M. abscessus* subspecies *bolletti* as independent species, rather than subspecies [6, 7]. Hence, the infections previously reported to be caused by *M. abscessus* can be *M*. *massiliense* or *M. bolletti.* The incubation period of RGM infection can range from 3 days to 7 months after acupuncture [8–10]. In our patient, the needles used in acupuncture remained in her body for about 2 years. For the treatment of *M. abscessus* subspecies *massiliense,* there are still no definite established optimal drugs, regimens, and duration of therapy [11]. According to ATS/ERS/ESCMID/IDSA 2020 guideline, the suggested regimen is macrolide-containing multidrug regimen that includes at least 3 active drugs guided by in vitro susceptibility. Thus, our patient received combination of 3 antibiotics including amikacin, clarithromycin, and ciprofloxacin. For the treatment duration of *M. abscessus* subspecies *massiliense,* a shorter duration or less intensive course of treatment may be sufficient and had better treatment outcome with reported median duration of 4.7 months of parenteral therapy and 12.1 months of total treatment course [11, 12].

*Scedosporium* species and related *Lomentospora prolificans* (formerly *Scedosporium prolificans*) are present in the environment where they play important roles in recycling of organic matter [13], commonly isolated from rural soils, polluted waters, composts, and from manure of cattle and fowl [14]. They have been increasingly recognized as causative agents of infections in immunocompromised patients [15]. Unfortunately, the identification of the species name of *Scedosporium* was not reachable, and we postulated that this pathogen opportunistically caused subcutaneous infection in our patient. To our knowledge, acupuncture-associated *Scedosporium* infection has never been reported. The management of infections due to Scedosporium depends on the underlying condition and voriconazole represents the first-line treatment. Moreover, the surgical resection is the key factor affect the successful infectious eradication [14].

In our patient, we think that acupuncture-associated *M. massiliense and Scedosporium* infections were already present in the acupuncture wounds and then superimposed by tetanus due to a recent history of injury to her shoulder and cultures growing both *M. massiliense and Scedosporium.* In addition, *M. massiliense* was also isolated from another acupuncture wound obtained from the abdominal wall.

## 4. Conclusions

To our knowledge, this is the first case of acupuncture-associated *M. massiliense* and *Scedosporium* infections superimposed by tetanus. In Thailand, the occurrence of acupuncture by nonqualified individuals and reemergence of tetanus remain prevalent. Hence, it is not uncommon to observe tetanus in association with acupuncture-related nontuberculous mycobacterial/fungal infection.

## Figures and Tables

**Table 1:** In vitro susceptibility and minimum inhibitory concentration (MIC) of *Mycobacterium massiliense*.

Antibiotic	Susceptibility	MIC (*μ*g/mL)
Cefoxitin	I	32
Amikacin	S	4
Clarithromycin (2 weeks after incubation)	S	0.12
Tigecycline	—	0.5
Linezolid	R	>32
Trimethoprim/sulfamethoxazole	R	>8
Moxifloxacin	R	8
Ceftriaxone	—	>64
Cefepime	—	>32
Imipenem	—	>64
Ciprofloxacin	R	>4
Minocycline	—	>8
Amoxicillin/clavulanate	—	>64
Tobramycin	R	8
Doxycycline	R	>16

S: susceptible, I: intermediate, and R: resistant.

## Abbreviations

dT: Diphtheria-tetanus
AFB: Acid-fast bacilli
TIG: Tetanus immunoglobulin
MIC: Minimum inhibitory concentration
RGM: Rapidly growing mycobacteria.
